# Irinotecan-containing doublet treatment versus irinotecan monotherapy as second-line choice for advanced gastric cancer

**DOI:** 10.1186/s12876-018-0772-4

**Published:** 2018-04-02

**Authors:** Liuting Yang, Xiaoyue Jiang, Han Yan, Yingying Li, Hongchao Zhen, Bingmei Chang, Seyed Kariminia, Qin Li

**Affiliations:** 1 Department of Biochemistry and Molecular Biology, Basic Medical College, Shanxi Medical University, Taiyuan, 030001 China; 2 Department of Oncology, Beijing Friendship Hospital, Capital Medical University, Beijing, 100050 China; 3 Department of Microbiology, Peking University Health Science Center, Beijing, 100190 China; 4 Department of Molecular and Cellular Oncology, MD Anderson Cancer Center, Houston, TX 77030 USA

**Keywords:** Irinotecan, Second-line chemotherapy, Advanced gastric cancer, Efficacy, Safety

## Abstract

**Background:**

For patients with advanced gastric cancer (AGC), second-line chemotherapy regimen remains controversial. The efficacy and safety of irinotecan-containing doublet treatment and irinotecan monotherapy were compared in this systematic analysis.

**Methods:**

A search was conducted on EMBASE and Medline databases. All articles compared irinotecan-containing doublet to irinotecan as second-line chemotherapy for AGC. STATA statistical software (Version 12.0) was used to analyze the data.

**Results:**

Seven studies, including 905 cases, were included in the analysis. Irinotecan-containing doublet treatment significantly prolonged progression-free survival compared to irinotecan monotherapy (HR = 0.82, 95% CI: 0.70–0.95). However, doublet treatment neither significantly prolong overall survival compared to monotherapy (HR = 0.94, 95% CI: 0.81–1.10), nor did it significantly increase the overall response rates and disease control rates, when compared to monotherapy. In addition, the irinotecan-containing doublet group had an increase in incidences of ≥ Grade 3 neutropenia (RR = 1.23, 95% CI: 1.01–1.51) and anemia (RR = 2.00, 95% CI: 1.37–2.92).

**Conclusions:**

When compared to irinotecan monotherapy, irinotecan-containing doublet treatment increased progression free survival and was tolerable as a second- line chemotherapy for AGC.

## Background

Gastric cancer ranks third in cancer-related mortality both worldwide and in China [1, 2]. Annually, there are over one million new cases of gastric cancer and 800,000 gastric cancer related deaths worldwide [1]. Even after radical surgery and adjuvant chemotherapy, the risk of local recurrence and distant metastasis remains high.

For patients with advanced gastric cancer (AGC), palliative chemotherapy could delay disease progression and increase the quality of life. Chemotherapy based on fluorouracil or platinum is the standard first-line therapy [3, 4]. Even after receiving first-line chemotherapy, the gastric cancer continues to progress and metastasize. Researchers reported that 20–70% patients with refractory AGC accepted the second-line treatment [5–7]. However, there are no standards or uniform guidelines about second-line chemotherapy regiments for AGC, and the optimal second-line chemotherapy choice remains controversial.

Several clinical trials showed irinotecan monotherapy, used as second-line treatment, significantly increased overall survival (OS) and progression-free survival (PFS), when compared to supportive care [8, 9]. Whether irinotecan-containing doublet is more beneficial than irinotecan monotherapy is a crucial question for researchers. However, the conclusions were not consistent. Higuchi K’s study reported that irinotecan combined with cisplatin improved PFS compared to irinotecan monotherapy and was more effective for PFS in AGC (PFS 3.8 versus 2.8 months, *P* = 0.039) [10] and for OS in intestinal-type AGC (OS 15.8 versus 14.0 months, *P* = 0.019) [11]. Satoh T’s study showed that in 5-fluorouracil-refractory AGC patients, irinotecan combined therapy was not superior to irinotecan monotherapy in terms of PFS. However, the study showed potential improvements in PFS and OS in the EGFR 2+/3+ subgroup (PFS, 118.5 versus 59.0 days; OS, 358.5 versus 229.5 days) [12]. Meanwhile, some studies have shown that irinotecan-containing doublet did not bring significant clinical benefit compared to irinotecan monotherapy [13, 14]. Therefore, we performed a systematic analysis to compare the efficacy and safety of irinotecan-containing doublet to irinotecan monotherapy as the second-line treatment for AGC.

## Methods

### Literature search plan

The systematic assessment was performed according to Preferred Reporting Items for Systematic Reviews and Meta-analysis criteria [15]. Two researchers (L.L. and Y.L.T) separately searched EMBASE and Medline. The keywords were “Gastric Cancer OR Gastric Neoplasm OR Gastric Carcinoma OR Gastric Cancers OR Gastroesophageal Junction Adenocarcinoma” AND “CPT-11 OR CPT 11 OR Irinotecan” AND “Refractory OR Second-line”.

The following were the inclusion criteria: (1) Phase II/III randomized controlled clinical trials (RCTs) or retrospective study; (2) Pathological diagnosis of AGC or gastroesophageal junction cancer; (3) ECOG scales 0 to 2; (4) First-line fluorouracil-containing chemotherapy failed; (5) Second-line treatment regimen was irinotecan-containing doublet versus irinotecan monotherapy.

### Data collection and quality assessment

The following information were included in this manuscript: first author, country, publication year, experimental design, number of patients, treatment regimen, hazard ratio (HR) and 95% confidence intervals (CIs) of OS and PFS, and the number of all grade/grades 3–4 adverse events. Two researchers (L.Y.Y and Z.H.C) assessed the risk of bias for individual studies according to Cochrane Collaboration’s tool. The following factors were evaluated: random sequence establishment, assignment concealment, blinding of subjects, blinding of results assessment and other bias. According to description of the text, each domain was divided into high risk, unclear risk and low risk.

### Data synthesis

STATA Statistical Software (Stata Corp., College Station, TX, USA, Version 12.0) was applied to conduct the analysis. The heterogeneity of each analysis was accessed by Chi-square test. The fixed effect model was applied when there was no heterogeneity between studies; on the contrary, the random effect model was used in the presence of heterogeneity. The combined effect of the overall response rate (ORR), disease control rate (DCR) and the incidences of toxicities were represented by risk ratio (RR). For ORR and DCR, RR > 1 indicated irinotecan-containing doublet was more effective than irinotecan monotherapy; for toxicities, RR > 1 indicated more adverse effect occurred in irinotecan-containing doublet group. To assess publication bias, Begg’s and Egger’s test were used.

## Results

### Selection of the trials

**Fig. 1:**
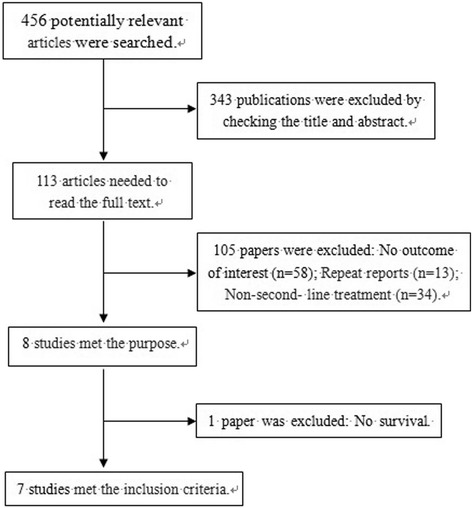
PRISMA flow diagram depicting the exclusion and inclusion of clinical trials in the systematic assessment

A total of 456 articles were obtained by searching the database initially. Three hundred forty-three publications were excluded by checking title and abstract. One hundred five articles were excluded after reading full text, due to no outcome of interest, repeat reports, and no second-line treatment. Finally, Seven studies [10–14, 16, 17] including 905 cases met the inclusion criteria. The characteristics of the including studies were shown in Table 1. The selective flow chart was shown in Fig. 1.

**Table 1:** Characteristics of the eligible studies included in the systematic assessment

Author and Year	Study design	Area	Study arms	Number of patients	mPFS(m)	mOS(m)	ORR(%)	DCR(%)
Higuchi K 2014 [10]	phase III clinical trial	Japan	irinotecan+cisplatin	64	3.8	10.7	22.0	75.0
			irinotecan	63	2.8	10.1	16.0	54.0
Nishikawa K 2015 [11]	phase III clinical trial	Japan	irinotecan+cisplatin	82	4.6	13.9	17.0	69.0
			irinotecan	81	4.1	12.7	16.0	65.0
Oba M 2011 [17]	retrospective study	Japan	irinotecan+cisplatin	42	2.7	9.8	20.0	54.3
			irinotecan	92	2.6	8	8.1	54.1
Satoh T 2015 [12]	phase II clinical trial	Japan and Korea	irinotecan+nimotuzumab	40	2.4	8.3	18.4	47.4
			irinotecan	43	2.8	7.7	10.3	46.2
Sym SJ 2013 [13]	phase II clinical trial	Korea	irinotecan+ 5-fluorouracil	30	3	6.7	20.0	56.7
			irinotecan	29	2.2	5.8	17.2	48.2
Tanabe K 2015 [14]	phase II/III clinical trial	Japan	irinotecan+S1	145	3.8	8.8	9.0	48.0
			irinotecan	148	3.4	9.5	9.0	53.0
Ueda A 2013 [16]	retrospective study	Japan	irinotecan+mitomycin C	22	3.9	9.6	19.0	86.0
			irinotecan	24	3.7	8.7	10.0	62.0

### Assessing risk of bias

**Fig. 2:**
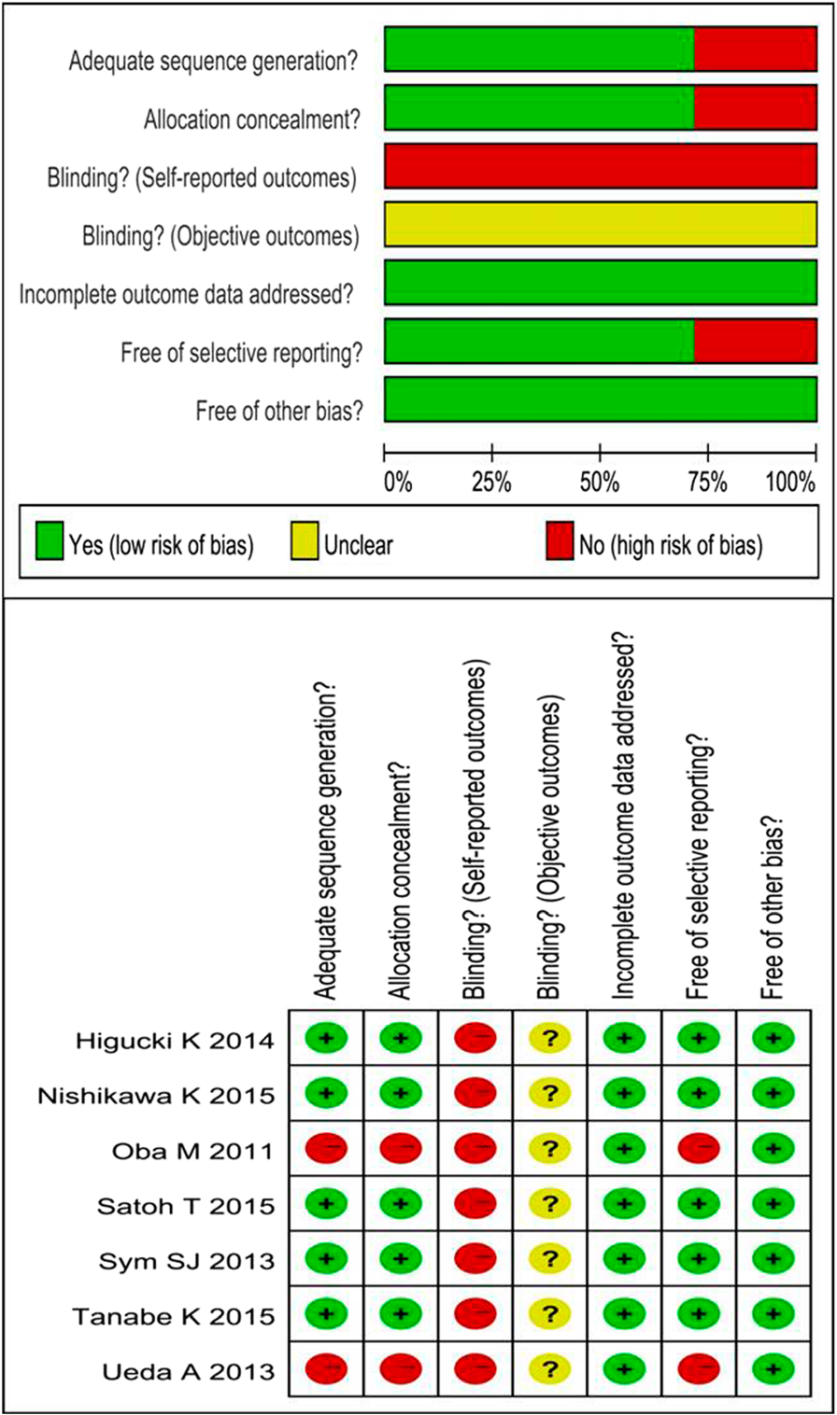
Risk of bias of the included studies

Cochrane risk-of-bias tool was used to assess the quality of the included study. The concrete contents of the risk-of-bias assessment were shown in Fig. 2. Two studies [16, 17] were retrospective, and the other five trials were open-labeled RCTs, which might negatively impact bias. Overall, the methodological quality of the studies was relatively satisfactory and fair.

### Efficacy

**Fig. 3:**
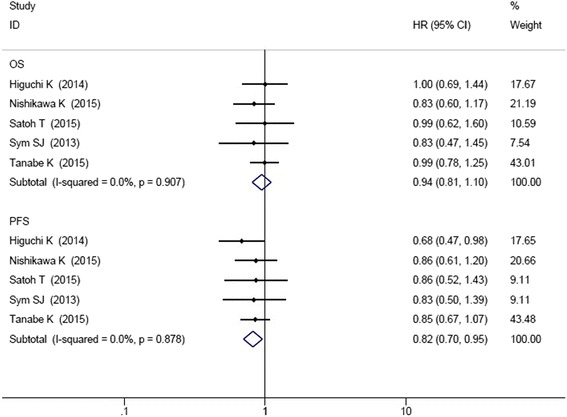
Comparison of OS and PFS between irinotecan-containing doublet versus irinotecan monotherapy

As shown in Fig. 3, five RCT studies reported the data of HR and 95% CIs for OS and PFS. There was no significant heterogeneity among the studies, so analysis was conducted by fixed effect model (OS: I^2^ = 0.0%, *P* = 0.907; PFS: I^2^ = 0.0%, *P* = 0.878). Meta-analysis demonstrated that irinotecan-containing doublet significantly prolonged PFS compared to irinotecan monotherapy (HR = 0.82, 95% CI: 0.70–0.95). The OS was not significantly improved in irinotecan-containing doublet group (HR = 0.94, 95% CI: 0.81–1.10).

**Fig. 4:**
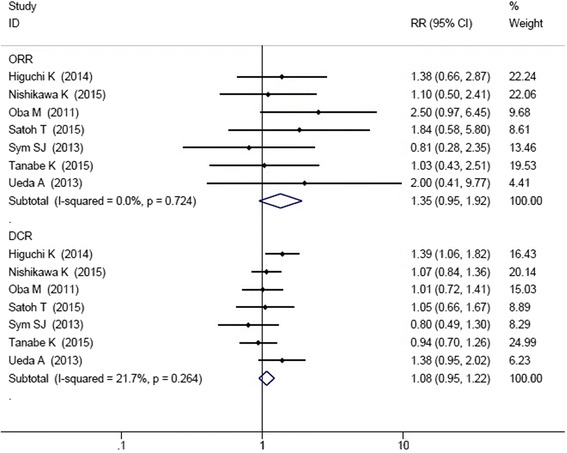
Comparison of ORR and DCR between irinotecan-containing doublet versus irinotecan monotherapy

All the seven studies reported the ORR and DCR. Chi-square test showed that I-squared was 0.0% for ORR and 21.7% for DCR, so the fixed effect model was applied. ORR and DCR were not significant improved in irinotecan-containing doublet group compared to irinotecan monotherapy group (ORR: HR = 1.35, 95% CI: 0.95–1.92; DCR: HR = 1.08, 95% CI: 0.95–1.22) (Fig. 4).

**Fig. 5:**
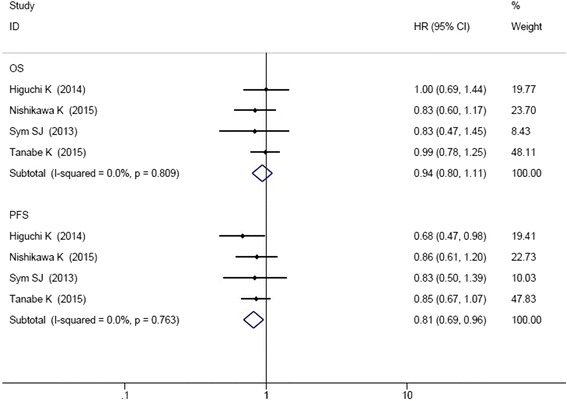
Comparison of OS and PFS between irinotecan combined cytotoxic chemotherapy versus irinotecan monotherapy

**Fig. 6:**
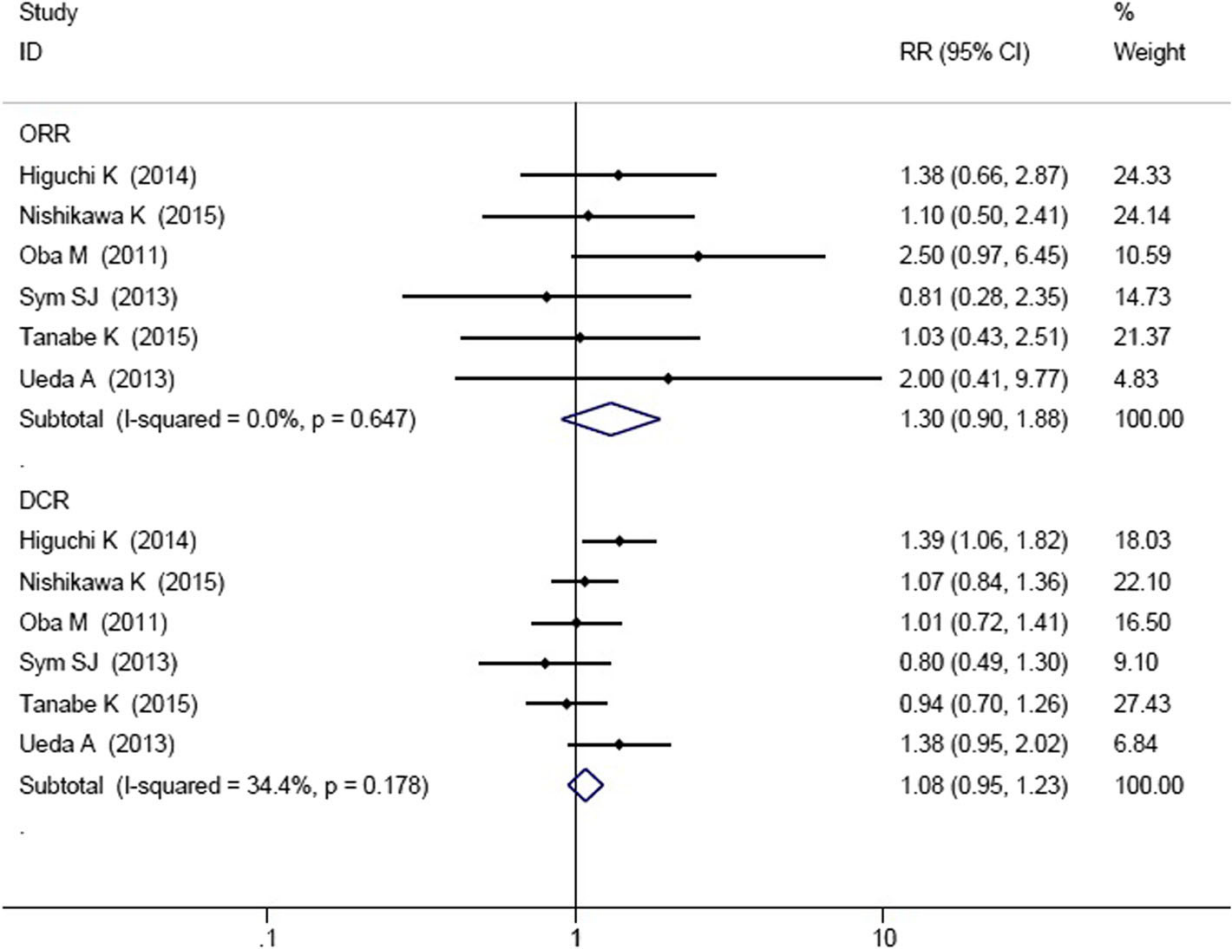
Comparison of ORR and DCR between irinotecan combined cytotoxic chemotherapy versus irinotecan monotherapy

In the seven studies, the combined regimens in six studies were irinotecan and cytotoxic chemotherapy. Therefore, we conducted the subgroup analysis of irinotecan plus cytotoxic chemotherapy versus irinotecan monotherapy. As shown in Fig. 5, four RCT studies reported the data of HR and 95% CIs for OS and PFS. The results showed that irinotecan-containing double cytotoxic chemotherapy significantly improved the PFS (HR = 0.81, 95% CI: 0.69–0.96). However, there were no significant differences in ORR (HR = 1.30, 95% CI: 0.90–1.88), DCR (HR = 1.08, 95% CI: 0.95–1.23), and OS (HR = 0.94, 95% CI: 0.80–1.11) between the two groups (Figs. 5 and 6).

### Safety

**Fig. 7:**
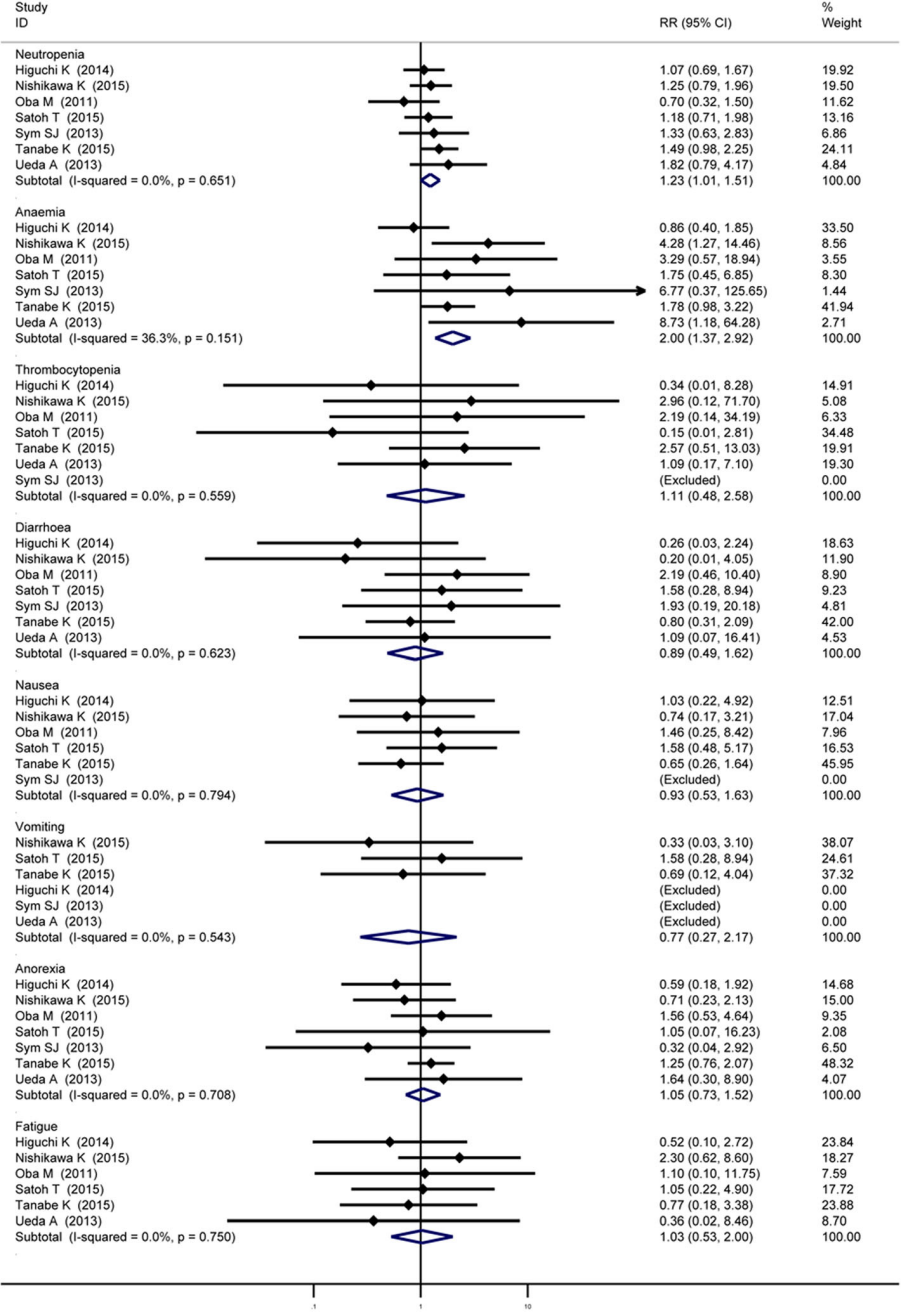
Comparison of ≥ Grade 3 toxicities between irinotecan-containing doublet versus irinotecan monotherapy

All the seven studies reported ≥ Grade 3 toxicities. In general, these adverse effects were manageable and tolerable in both groups. Since there were no significant heterogeneities among the studies, the meta-analysis was conducted by fixed effect model. The incidences of ≥ Grade 3 neutropenia (RR = 1.23, 95% CI: 1.01–1.51) and anemia (RR = 2.00, 95% CI: 1.37–2.91) were significantly increased in irinotecan-containing doublet group when compare to irinotecan monotherapy group. For other ≥ Grade 3 toxicities, the differences between the two groups were not significant. The related results were shown in Fig. 7 and Table 2.

**Table 2:** Comparison of ≥ Grade 3 toxicities rates between irinotecan-containing doublet versus irinotecan monotherapy

Adverse events	Irinotecan-containing doublet event/total (%)	Irinotecan monotherapy event/total (%)
Neutropenia	142/425 (33.4)	128/483 (26.5)
Anemia	68/425 (16.0)	36/483 (7.45)
Thrombocytopenia	9/395 (2.3)	9/454 (2.0)
Diarrhea	17/425 (4.0)	22/483 (4.6)
Nausea	21/373 (5.6)	25/430 (5.8)
Vomiting	6/267 (2.2)	8/272 (2.9)
Anorexia	47/425 (11.0)	50/483 (10.4)
Fatigue	16/395 (4.1)	17/454 (3.7)

### Publication bias

There were no publication bias for OS (Egger’s test, *P* = 0.530; Begg’s test, *P* = 0.462) and PFS (Egger’s test, *P* = 0.760; Begg’s test, *P* = 0.462). Similarly, there were no publication bias for the ORR (Egger’s test, *P* = 0.546; Begg’s test, *P* = 1.000) and DCR (Egger’s test, *P* = 0.503; Begg’s test, *P* = 0.548).

## Discussion

As second-line treatment, whether irinotecan-containing doublet is more beneficial than irinotecan monotherapy for AGC has been a controversial question. Regarding this topic, our meta-analysis showed that irinotecan-containing doublet did not significantly prolong OS or improve ORR and DCR compared to irinotecan monotherapy. However, PFS was significantly prolonged in the combined treatment group. Meanwhile, subgroup analysis showed the similar results. These results were not very consistent with previous results reported by Zhang et al., which stated that cytotoxic doublet chemotherapy did not significantly benefit PFS, OS and ORR when compared to cytotoxic monotherapy [18].

Despite the negative results of OS, there was significant improvement of PFS brought by the irinotecan-containing doublet treatment. There are many factors causing the results: (1) The treatment regimens in the seven studies were not identical, which included irinotecan plus cisplatin/5-FU/S1/ mitomycin C/nimotuzumab. The synergistic and antagonistic effects of combined treatment may lead to different efficacy. Studies reported that irinotecan acted synergistically with cisplatin/5-FU/mitomycin C, which might contribute to the extension of PFS [19, 20]. (2) Some patients enrolled received the post-treatments, which might affect OS but not PFS. (3) The pathologic subtypes might influence the results. Irinotecan combined with cisplatin was reported to be more effective for intestinal-type gastric cancer [11], which gave us a clue that the researches based on pathological stratification might bring more accurate results. (4) The Karnofsky score for the patients at enrollment might influence the results. The patients in good physical condition might take the full treatment and tolerate more toxic side effects, which might bring benefit for PFS. (5) Ethnic and regional differences might influence the results. In the ToGA study, trastuzumab markedly influenced the survival of patients in South America, despite the fact that more than 50% of study subjects were Korean and Japanese patients [21]. Similarly, in the AVAGAST study, bevacizumab did not influence the survival in Asia, but it markedly improved survival in the Pan-Americans [22]. All the data in our meta-analysis come from Japan and Korea, so the result had one-sidedness and data from other regions is worth anticipated. (6) Biomarker-directed therapy is very important for efficacy and safety [23]. The patients with UGT1A1*6 and UGT1A7*3 were prone to severe neutropenia, and the patients with UGT1A9*1b were prone to severe diarrhea. Biomarkers will help distinguish the optimal patients receiving irinotecan. (7) The small sample size of several studies might result in inadequate statistical power to find the OS difference.

In our study, only one research involved the combination of targeted agents and irinotecan [12]. There was no superiority of PFS when nimotuzumab combined with irinotecan compared to irinotecan alone in 5-fluorouracil-refractory AGC patients. However, the combination therapy significantly improved PFS and OS in the EGFR 2+/3+ subgroup [12]. The precise of pathological stratification is manifested once again. Recent studies showed that ramucirumab was superior to the best supportive care in AGC [24], and a double-blind, phase III RCT revealed the ramucirumab combined with paclitaxel significantly improved OS and PFS when compared to paclitaxel alone as second-line choice for AGC [25]. These results indicate that more clinical trials involving targeted agents combined with irinotecan versus irinotecan monotherapy are needed.

The adverse effects of chemotherapy were inevitable. A complete evaluation of chemotherapy should integrate anti-tumor effects and adverse reactions. Our meta-analysis showed irinotecan combined regimen significantly increased the incidence of neutropenia and anemia, but it had no significant influence on the incidence of thrombocytopenia, diarrhea, nausea, vomiting, anorexia and fatigue. Neutropenia is controllable during chemotherapy. However, the physical condition of the patient before combined chemotherapy is still critical. UGT1A1*6, UGT1A7*3 and UGT1A9*1b should be monitored regularly to avoid the incidence of the severe neutropenia and diarrhea. Anemia, especially severe anemia, is relatively a more serious problem than neutropenia, so the patients with anemia should be cautious while receiving irinotecan-containing doublet treatment. An appropriate dose of irinotecan could reduce the incidences of toxic effects.

## Conclusions

In summary, irinotecan-containing doublet improved PFS compared to irinotecan monotherapy, and it was tolerable as second-line chemotherapy for AGC. However, it did not demonstrate an OS benefit. There is an urgent need for predictive biomarkers and pathological classification to guide the selection of patients. The optimal time, dosage and population are also important to explore the real therapeutic value of irinotecan-containing doublet treatment. Large-scale RCT studies will provide more precise answers.

## Abbreviations

AGC: Advanced gastric cancer
DCR: Disease control rate
EGFR: Epidermal growth factor receptor
EMBASE: Excerpta medica database
HR: Hazard ratio
OR: Odds ratio
ORR: Overall response rate
OS: Overall survival
PFS: Progression-free survival
PRISMA: Preferred reporting items for systematic reviews and meta-analysis
RCT: Randomized controlled trial
RR: Risk ratio

## References

[1] Fitzmaurice C, Allen C, Barber RM, et al. Global, regional, and national cancer incidence, mortality, years of life lost, years lived with disability, and disability-adjusted life-years for 32 cancer groups, 1990 to 2015: a systematic analysis for the global burden of disease study. JAMA Oncol. 2017;3:524–48. 10.1001/jamaoncol.2016.5688 PMC6103527 27918777

[2] Chen W, Zheng R, Zeng H, Zhang S, He J (2015). Annual report on status of cancer in China, 2011. Chin J Cancer Res.

[3] Boku N, Yamamoto S, Fukuda H (2009). Fluorouracil versus combination of irinotecan plus cisplatin versus S-1 in metastatic gastric cancer: a randomised phase 3 study. Lancet Oncol.

[4] Van Cutsem E, Moiseyenko VM, Tjulandin S (2006). Phase III study of docetaxel and cisplatin plus fluorouracil compared with cisplatin and fluorouracil as first-line therapy for advanced gastric cancer: a report of the V325 study group. J Clin Oncol.

[5] Elsing C, Herrmann C, Hannig CV, Stremmel W, Jäger D, Herrmann T (2013). Sequential chemotherapies for advanced gastric cancer: a retrospective analysis of 111 patients. Oncology.

[6] Catalano V, Graziano F, Santini D (2008). Second-line chemotherapy for patients with advanced gastric cancer: who may benefit. Br J Cancer.

[7] Lee J, Lim T, Uhm JE (2007). Prognostic model to predict survival following first-line chemotherapy in patients with metastatic gastric adenocarcinoma. Ann Oncol.

[8] Kang JH, Lee SI, Lim DH (2012). Salvage chemotherapy for pretreated gastric cancer: a randomized phase III trial comparing chemotherapy plus best supportive care with best supportive care alone. J Clin Oncol.

[9] Thuss-Patience PC, Kretzschmar A, Bichev D (2011). Survival advantage for irinotecan versus best supportive care as second-line chemotherapy in gastric cancer--a randomised phase III study of the Arbeitsgemeinschaft Internistische Onkologie (AIO). Eur J Cancer.

[10] Higuchi K, Tanabe S, Shimada K (2014). Biweekly irinotecan plus cisplatin versus irinotecan alone as second-line treatment for advanced gastric cancer: a randomised phase III trial (TCOG GI-0801/BIRIP trial). Eur J Cancer.

[11] Nishikawa K, Fujitani K, Inagaki H (2015). Randomised phase III trial of second-line irinotecan plus cisplatin versus irinotecan alone in patients with advanced gastric cancer refractory to S-1 monotherapy: TRICS trial. Eur J Cancer.

[12] Satoh T, Lee KH, Rha SY (2015). Randomized phase II trial of nimotuzumab plus irinotecan versus irinotecan alone as second-line therapy for patients with advanced gastric cancer. Gastric Cancer.

[13] Sym SJ, Hong J, Park J (2013). A randomized phase II study of biweekly irinotecan monotherapy or a combination of irinotecan plus 5-fluorouracil/leucovorin (mFOLFIRI) in patients with metastatic gastric adenocarcinoma refractory to or progressive after first-line chemotherapy. Cancer Chemother Pharmacol.

[14] Tanabe K, Fujii M, Nishikawa K (2015). Phase II/III study of second-line chemotherapy comparing irinotecan-alone with S-1 plus irinotecan in advanced gastric cancer refractory to first-line treatment with S-1 (JACCRO GC-05). Ann Oncol.

[15] Moher D, Liberati A, Tetzlaff J, Altman DG (2009). Preferred reporting items for systematic reviews and meta-analyses: the PRISMA statement. BMJ.

[16] Ueda A, Hosokawa A, Ogawa K (2013). Non-randomized comparison between irinotecan plus mitomycin C and irinotecan alone in patients with advanced gastric cancer refractory to fluoropyrimidine and platinum. Anticancer Res.

[17] Oba M, Chin K, Kawazoe Y (2011). Irinotecan monotherapy offers advantage over combination therapy with irinotecan plus cisplatin in second-line setting for treatment of advanced gastric cancer following failure of fluoropyrimidine-based regimens. Oncol Lett.

[18] Zhang Y, Ma B, Huang XT, Li YS, Wang Y, Liu ZL (2016). Doublet versus single agent as second-line treatment for advanced gastric cancer: a meta-analysis of 10 randomized controlled trials. Medicine (Baltimore).

[19] Kano Y, Suzuki K, Akutsu M (1992). Effects of CPT-11 in combination with other anti-cancer agents in culture. Int J Cancer.

[20] Satoh H, Ohtomo M, Ishikawa H (2002). In vivo advantage of combined administration with CPT-11 and 5-fluorouracil in rats. J Exp Ther Oncol.

[21] Bang YJ, Van Cutsem E, Feyereislova A (2010). Trastuzumab in combination with chemotherapy versus chemotherapy alone for treatment of HER2-positive advanced gastric or gastro-oesophageal junction cancer (ToGA): a phase 3, open-label, randomised controlled trial. Lancet.

[22] Ohtsu A, Shah MA, Van Cutsem E (2011). Bevacizumab in combination with chemotherapy as first-line therapy in advanced gastric cancer: a randomized, double-blind, placebo-controlled phase III study. J Clin Oncol.

[23] Cui C, Shu C, Cao D (2016). UGT1A1*6, UGT1A7*3 and UGT1A9*1b polymorphisms are predictive markers for severe toxicity in patients with metastatic gastrointestinal cancer treated with irinotecan-based regimens. Oncol Lett.

[24] Fuchs CS, Tomasek J, Yong CJ (2014). Ramucirumab monotherapy for previously treated advanced gastric or gastro-oesophageal junction adenocarcinoma (REGARD): an international, randomised, multicentre, placebo-controlled, phase 3 trial. Lancet.

[25] Wilke H, Muro K, Van Cutsem E (2014). Ramucirumab plus paclitaxel versus placebo plus paclitaxel in patients with previously treated advanced gastric or gastro-oesophageal junction adenocarcinoma (RAINBOW): a double-blind, randomised phase 3 trial. Lancet Oncol.

